# Phase, Composition and Structure Changes of CoCrNi-Based Concentrated Alloys Resulting from High Temperature Oxidation

**DOI:** 10.3390/ma13102276

**Published:** 2020-05-15

**Authors:** Monika Vilémová, Hynek Hadraba, Zdeněk Weiss, František Lukáč, Štefan Csáki, Zdeněk Chlup, Jiří Matějíček, Tomáš Chráska

**Affiliations:** 1 Department of Materials Engineering, Institute of Plasma Physics of the CAS, Za Slovankou 1782/3, 182 00 Prague, Czech Republic; lukac@ipp.cas.cz (F.L.); csaki@ipp.cas.cz (Š.C.); matejicek@ipp.cas.cz (J.M.); tchraska@ipp.cas.cz (T.C.); 2 Institute of Physics of Materials, Czech Academy of Sciences, Žižkova 22, 616 00 Brno, Czech Republic; hadraba@ipm.cz (H.H.); chlup@ipm.cz (Z.C.); 3 Institute of Physics of the Czech Academy of Sciences, Na Slovance 2, 182 21 Prague, Czech Republic; weissz@fzu.cz

**Keywords:** FeCoNiCr, CrMnFeCoNi, high-entropy alloys, medium-entropy alloys, Spark Plasma Sintering, Y-Ti complex oxide nanoparticles, Y_2_O_3_ particles, oxide dispersion strengthening, passivation, Cr_23_C_6_ dissolution

## Abstract

In this work, CoCrNi, FeCoCrNi and CoCrFeMnNi concentrated alloys with a Y-Ti oxide particle dispersion were prepared by mechanical alloying and Spark Plasma Sintering. The alloy consists of an FCC Ni-based matrix with a Y-Ti oxide dispersion and additional phases of Cr_23_C_6_ and Cr_2_O_3_. The effect of Fe, Mn, and Y-Ti oxide particles on the formation of oxide scales and the composition of the adjacent CoCrNi and FeCoCrNi alloys was studied. It was found that alloys without Mn in their composition form a protective Cr_2_O_3_ scale. The Cr_23_C_6_ particles provide an alternative mechanism for balancing the chromium loss during the oxidation. Y and Ti from the oxide particles participate in the formation of the protective oxide scales. Fe promotes Y and especially Ti diffusion through the Cr_2_O_3_ scale, resulting in the formation of Ti-depleted regions in the alloy. The findings will serve for the further development of these new materials.

## 1. Introduction

Conventional materials fail to satisfy requirements in a number of advanced applications. Among the future technologies “waiting” for new materials and which are delayed due to the unsatisfactory performance of available alloys are nuclear fusion reactors, generation IV fission reactors, turbine engines, and hydrogen-fueled technologies. Even though each of the applications needs a specific set of properties, the requirement of good corrosion resistance remains common for all of them. In some cases, such as turbines, the application of a protective coating is possible; in others, corrosion resistance must be an inherent material property. 

The development of so-called high-entropy alloys (HEAs) was driven by interest in exploring the central regions of multicomponent phase diagrams and resulted in the finding that such a concentrated material composition might lead to single-phase solid solution instead of the expected number of intermetallic phases [1]. Moreover, HEAs showed interesting and sometimes contradictory properties, such as high hardness and superior plasticity. The exceptional mechanical performance of HEAs seems suitable for several future applications; however, their use might be limited, since many applications require balanced properties. Therefore, there is an interest in enhancing the unsatisfactory properties such as oxidation resistance. Various ways of improvement have been explored; these include additional alloying [2], the introduction of additional phases [3,4], or eliminating some elements at the expense of the entropy state while keeping the composition in the central regions of phase diagrams [5].

Most of the studied Ni-containing HEAs consist of mainly 3D transition metals. In a broad view, they belong to the family of Cantor alloys, i.e., CoCrFeMnNi derivatives [3,5,6]. The oxidation behavior of nickel-, iron-, and cobalt- containing alloys was extensively studied in systems with two major elements. Chromium is frequently the oxide-forming element in the alloys, and their oxidation behavior is strongly dependent on the chromium content. Nickel-based alloys with Cr exceeding 10 wt. % develop a passivating layer of outer Cr_2_O_3_ scale at up to 1000 °C [7]. For iron–chromium alloys, passivation is achieved above 20 wt. % of Cr [7]. Nevertheless, at high temperatures the oxide scale is not protective, as iron ions diffuse through the Cr_2_O_3_ scale [7]. Cobalt alloys gain oxidation resistance at above 30 wt. % of Cr. Studies on the oxidation performance of HEAs have emerged in recent years. A few papers have shown that, despite the relatively high Cr content, the oxidation performance of CoCrNiMnFe (18.5 wt. % Cr) HEA above 600 °C is extremely poor. In the original equiatomic composition of the alloy, the oxide scale consists of several layers containing chromium oxide, manganese oxides, and iron oxide [8,9]. The deteriorating effect was assigned to the Mn addition and was observed also for CoCrNiMn (23 wt. % Cr) alloy at 800–1000 °C [10]. Moreover, in systems with high Mn diffusion rates, Mn-based scales might lead to the formation of subsurface porosity in the bulk alloy [11]. Thus, several studies have focused on limiting the Mn content, and a significant improvement in oxidation behavior was observed [10,12]. Alternatively, Wang et al. [13] proved that the oxidation resistance of CoCrNiMnFe can be improved by the introduction of plastic deformation. Attempts to increase oxidation performance by alloying with Al were also successful [14]. 

Recent studies prove that exceptional mechanical properties can be achieved even for medium-entropy alloys such as CoCrNi and FeCoCrNi, especially when coupled with particle dispersion [5,15,16]. Y_2_O_3_ is the most frequently used oxide for particle dispersion due to its high temperature stability. Y_2_O_3_ may be enriched by Ti; this has been shown to further increase the high temperature resistance and radiation damage resistance [17]. Another proven effect of yttrium addition is the enhancement of oxidation resistance [18]. Thus, with the need to find suitable materials for advanced applications, it is necessary to study and understand the overall high-temperature behavior of new multi-component materials with concentrated compositions.

So far, the majority of oxidation studies have been performed on cast or homogenized materials. The formation of dendritic structures, the segregation of elements in interdendritic regions, and large grain sizes are typical for such processing methods. In the presented work, CoCrNi, FeCoCrNi, and CoCrFeMnNi alloys with a Y-Ti-O particle dispersion were prepared in the form of powders by the mechanical alloying route. Mechanical alloying produces a homogeneous distribution of the elements in the alloy and allows the dispersion of oxide particle-forming elements. The powders were compacted by Spark Plasma Sintering, i.e., technology that hinders excessive grain growth and allows the precipitation of oxide particles. The research aimed to study the effect of Fe and Mn coupled with Y-Ti oxide particles on the formation of oxide scales and the composition of adjacent equiatomic CoCrNi and FeCoCrNi alloys. The results were compared with the results of CoCrFeMnNi high-entropy alloy, which served as a reference.

## 2. Materials and Methods 

The CoCrNi, FeCoCrNi and CoCrFeMnNi equiatomic alloys containing a Y-Ti-O particle dispersion were prepared by a mechanical alloying process from elemental powders (Sigma Aldrich, St. Louis, MO, USA); purity in wt. %: Cr > 99%, Co > 99.9%, Fe > 99%, Mn > 99%, Ni > 99.7%, Y > 99.5%, Ti > 99.7%). The 100 g blend of powders was mechanically alloyed using a planetary ball mill (Pulverisette P-6, Fritsch, Idar-Oberstein, Germany). The milling process was carried out in a hardened steel vial with steel balls for 24 h under vacuum. The ball to powder ratio was 15:1 and a rotational speed of 350 rpm was used. The oxides were introduced by adding O_2_ gas, Y (0.24 wt. %), and Ti (0.3 wt. %) to the blend.

The alloyed powders were compacted using a SPS 10-4 Spark Plasma Sintering machine (Thermal Technology LLC, Santa Rosa, CA, USA) at a temperature of 1150 °C, pressure of 50 MPa, 5 min dwell time, and a heating/cooling rate of 100 °C/min. The identification of crystalline phases in the base material and in the oxide scales was done by X-ray diffraction (XRD). The measurements were carried out with a D8 Discover diffractometer (Bruker AXS, Karlsruhe, Germany) using CoKα radiation and a 1D LynxEye detector (Fe β filter in front of the detector). A divergent X-ray beam was aimed at the samples’ surfaces before and after oxidation; a parallel x-ray beam with a diameter of 1 mm was formed by a polycapillary unit and used for the phase analysis of the samples on the cross-section in the area below the oxide scale. The subsequent quantitative Rietveld refinement analysis [19] was performed with TOPAS V5 software (Bruker, Karlsruhe, Germany) [20].

The melting point of the prepared material was determined using a differential thermal analysis (DTA) with a heating rate of 10 °C/min under the flow of Ar. Two individual measurements were taken on samples (cut from the samples prepared by the SPS) of about 30 mg which were placed in an alumina crucible. The melting point was determined as the onset temperature of the melting-induced peak. Finally, the average value of the two determined temperatures was used.

Oxidation experiments were performed in a tube furnace at 750 °C and 950 °C using synthetic air (20.9% O_2_, 78.8% N_2_, 3 ppm H_2_O) within a time range of 1 to 150 h. The samples were ground using SiC papers up to FEPA P1200 and cleaned in acetone prior to oxidation. The heating rate up to the oxidation temperature was 5 °C/min; cooling to 700 °C was conducted at a controlled rate of 5 °C/min, and then the furnace was freely cooled down to room temperature. After each oxidation cycle, the samples were weighted on an analytical scale. A negligibly small spallation occurred for CoCrNi and FeCoCrNi; this was not included in the weight-gain plots. For CoCrFeMnNi, the spallation was significant, however it was not included in the kinetic curves either in order to demonstrate the dramatic failure of the scale. 

The elemental composition of the non-oxidized samples was measured by X-ray fluorescence (XRF) using a Bruker S2 PUMA spectrometer (Bruker, Madison, WI, USA) under vacuum.

The concentration–depth distributions of the elements in the oxide scales and adjacent alloy were established by Glow discharge optical emission spectroscopy (GD-OES) [21]. The analyses were performed using the GDA750 HR spectrometer (Spectruma GmbH., Hof, Germany) in a DC discharge in argon with a 2.5 mm-diameter anode at 800V and 15mA. The quantitative analysis was based on a sputter rate-corrected calibration [22], with certified reference materials of CrNi and CrMn steels and Ni and Co alloys. The calibration functions for yttrium were established by in-house set-up samples of MgY alloys, and the calibration function of oxygen was based on the stoichiometric FeO and NiO layers.

## 3. Results and Discussion

**Figure 1:**
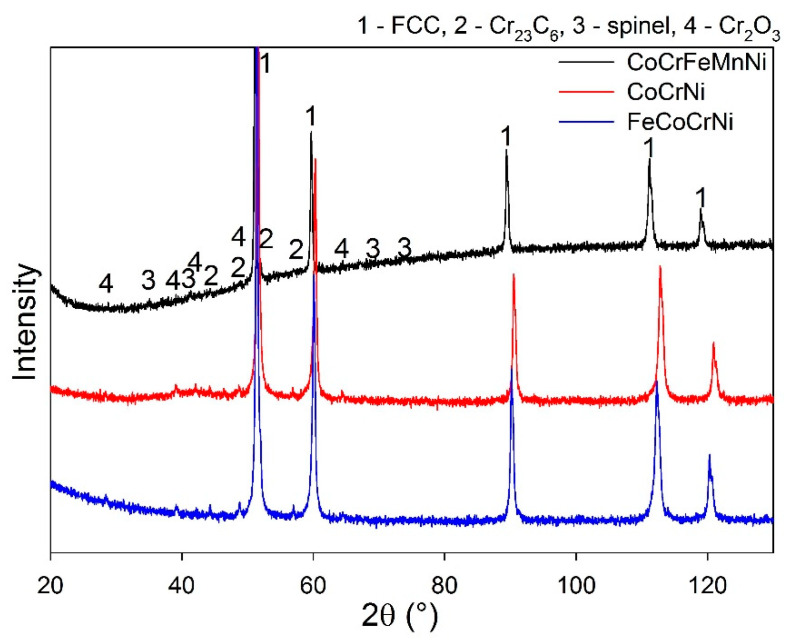
Phase composition of the prepared alloys showing a single-phase FCC matrix lattice for all alloys; the CoCrNi and FeCoCrNi contain additional Cr_23_C_6_ and Cr_2_O_3_ phases, and the CoCrFeMnNi also contains a spinel phase. The peak positions are designated by numbers referring to the legend in the top right corner.

The elemental composition of the sintered alloys is summarized in Table 1. The XRD results show that all the prepared alloys have an FCC nickel-based matrix and contain additional Cr_23_C_6_ and Cr_2_O_3_ phases (Figure 1, Table 2). A possible source of carbon is the graphite die used for sintering. The oxides likely stem from inherent oxygen impurities in the Cr powder and the introduction of O_2_ gas during milling; minor oxygen impurities might be gained also during the sintering process, where the vacuum atmosphere is limited to 5 Pa. The spinel oxide structure is likely a Mn-based oxide, since the phase is observed only in the CoCrFeMnNi alloy. The lattice parameter is close to Mn_1.7_Cr_1.3_O_4_ (a = 8.424 Å). A small volume of fine nano-crystalline Y-Ti oxides distributed within a large volume of the HEA matrix is below the XRD detection limit; thus, weak diffraction peaks of the oxides are not visible in the measured spectra. The presence of the Y-Ti oxides was, nevertheless, proved in the authors’ previous study by TEM and EDS [16]. The literature reports that Y_2_TiO_5_ and Y_2_Ti2O_7_ are the most common oxides present in Y-Ti-doped alloys [23]. The phase content of the prepared alloys correlates with the data in the literature. A single-phase FCC matrix is reported for CoCrFeMnNi [10,14] as well as for CoCrNi prepared by melting and subsequent thermo-mechanical post-processing [24,25]. The presence of carbide phases depends on the amount of C intake. The carbon solubility in CoCrNi alloys is as low as 0.67 at. % [26] and, above this limit, M_23_C_6_ carbide-type is formed.

**Figure 2:**
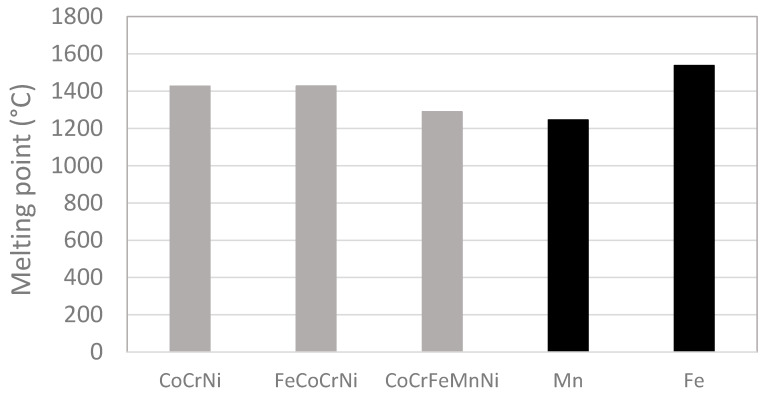
The effect of Mn and Fe on the melting point of the CoCrNi-based alloys. The melting point of Mn and Fe (black columns) was plotted for a comparison with the produced alloys (grey columns).

Figure 2 shows the variation of the melting point among the prepared alloys. While Mn causes a significant decrease in an alloy’s melting point, Fe has no effect on the melting point of CoCrNi. Although the melting point of CoCrNi and FeCoCrNi was increased by the elimination of Mn, it is only slightly above that of commercial nickel-based alloys [27], and below that of pure nickel (1455 °C). Cr and Co are a common addition to nickel-based alloys. It is known that Cr causes a decrease in the melting point, whereas Co has an opposite effect. However, the change is very weak and in the range of 0–30 at. % [28]. Thus, to further increase the melting point, the introduction of a new element seems necessary.

With respect to the increased melting point compared toCoCrFeMnNi, the alloys were tested at two temperatures, i.e., 750 °C and 950 °C. Even though the higher tested temperature was still well below the melting point of CoCrNi and FeCoCrNi, values lower than 1000 °C were chosen to prevent excessive oxide volatilization in the case of Cr_2_O_3_ formation [29]. The phase stability of the alloys was also considered. Based on the research performed on CoCrFeMnNi by Park et al. and Shuh et al. [30,31], it is well known that the short-term stability of the single solid solution is assured at temperatures higher than 700 °C; therefore, both temperatures were situated above this limit. 

**Figure 3:**
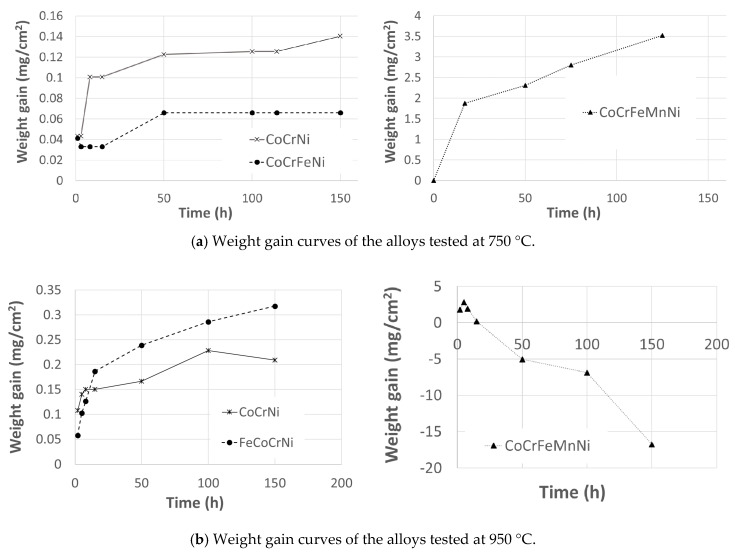
Oxidation kinetics represented by cumulative weight gain within 150 h of air exposure at high temperatures. Major spallation was observed only for CoCrFeMnNi at 950 °C, and the debris were not included in the measured weight.

Figure 3a shows a comparison of the oxidation performance of the tested alloys at 750 °C. For CoCrFeMnNi, two-stage oxidation behavior is observed. The alloy does not form an effective oxidation barrier, as the oxidation rate in the second stage does not decrease with the oxide thickness. However, after 150 h at 750 °C, no major oxide spallation was observed. Removing manganese from the alloy composition significantly decreased the weight gain of FeCoCrNi, and the oxide provides a protective barrier against further oxidation. The results agree with [12], where the mass gain reached comparable values even though the alloy composition included a small amount of Mn. Further restriction of the composition to CoCrNi leads to a mild increase in the weight gain; however, the kinetics remained similar to FeCoCrNi. 

**Figure 4:**
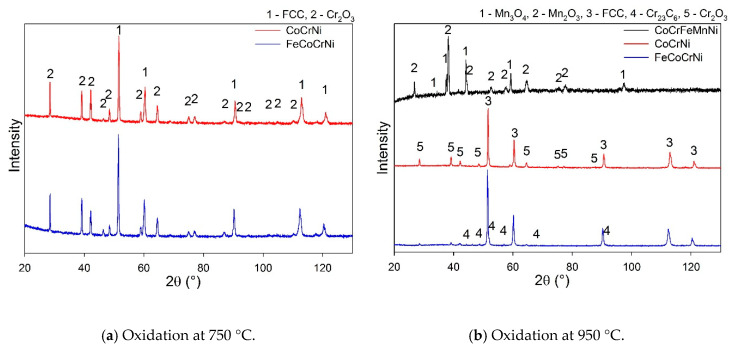
Phase analysis of the oxide scales formed on the alloys after 150 h of oxidation. The quantitative analysis of the phases is summarized in Table 2.

Phase analysis of CoCrFeMnNi confirmed the formation of Mn oxides (Figure 4a, Table 2). According to the Ellingham diagram, MnO has a lower Gibbs free energy of formation than Cr_2_O_3_. MnO was found in the authors’ previous study to be the initial oxide layer formed on the alloy’s surface [11]. Moreover, Mn has the highest diffusion coefficient in the CoCrFeMnNi alloy among all the constituents [32] which, coupled with the non-protective character of the Mn oxides, must lead to the depletion of the alloy surface of Mn. Both the FeCoCrNi and CoCrNi alloys form a protective Cr_2_O_3_ scale. The weight gain values (Figure 3a) suggest that the oxide scale is very thin, which explains the presence of a Ni-based matrix in the spectra (Figure 4a). Furthermore, the carbide phase disappeared from the CoCrNi alloy diffractogram (Figure 4a, Table 2). It can thus be expected that the carbides were somehow involved in the process of oxidation.

The oxide scale on CoCrFeMnNi developed major spallation after 5 h at 950 °C. This was reflected in a large drop in the weight gain curve (Figure 3b). FeCoCrNi showed two-stage oxidation behavior. In the first stage, the fast growth of the oxide is apparent; in the second stage, the oxide scale gained a protective character and the oxidation rate decreased with increasing oxide thickness. More complex behavior was reported in [33], where arc-melted FeCoCrNi showed three stages within 100 h of oxidation and the weight gain was already higher during the first stage. Although the alloys were prepared from high-purity powders, the very distinct microstructure containing dendritic arms is likely responsible for the different kinetics of oxide scale formation. Disregarding the different phases of the oxidation kinetics, removing Mn from the alloy composition leads to an improvement in the scale integrity and therefore to the improved protection of the alloy (Figure 3b, FeCoCrNi). The behavior of CoCrNi is similar to FeCoCrNi. In the first stage, the oxide layer is formed rapidly; in the second stage, the weight gain decreases and is lower than in the case of FeCoCrNi. Clearly, the addition of Fe to CoCrNi increases the weight gain during the oxidation at 950 °C; however, the protective character of the scale seems to be preserved within 150 h of oxidation. The larger weight gain of FeCoCrNi is likely to be caused by an Fe ion diffusion that has a high solubility in the Cr_2_O_3_ scale and might lead to the formation of an iron oxide outer scale after long time periods [7].

The XRD analysis was performed only on CoCrNi and FeCoCrNi, as the scale on CoCrFeMnNi was spalled off. The XRD spectra proved the formation of Cr_2_O_3_ scale on both alloys (Figure 4b, Table 2). Additionally, after oxidation at 950 °C, carbide peaks disappeared from the matrix reflections of CoCrNi as well as of FeCoCrNi.

To explain the processes related to the formation of oxide scale, an in depth elemental analysis of the alloy was performed by Glow discharge optical emission spectroscopy (GDOES). The analysis was performed only for FeCoCrNi and CoCrNi; CoCrFeMnNi was not analyzed due to the formation of a nonconductive scale in the case of 750 °C oxidation and to massive spallation in the case of 950 °C oxidation. Instead, an EDS analysis was performed for CoCrFeMnNi tested at 750 °C. However, the EDS resolution is not sufficient to quantitatively analyze low-Z elements, such as C, and low concentration elements, such as Ti and Y. 

**Figure 5:**
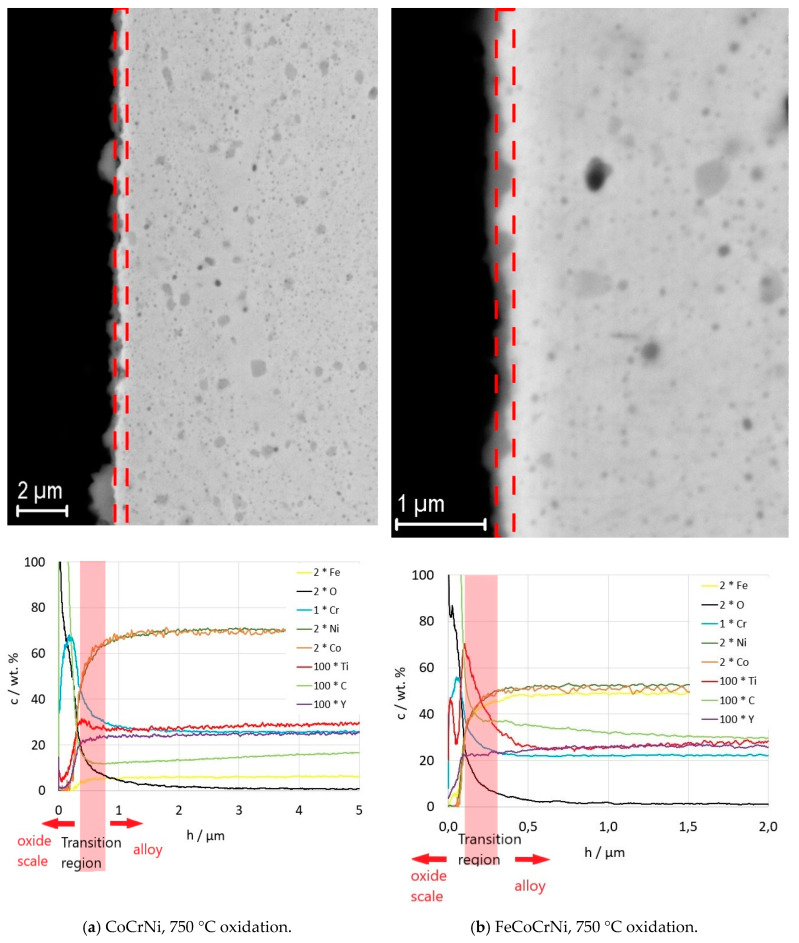

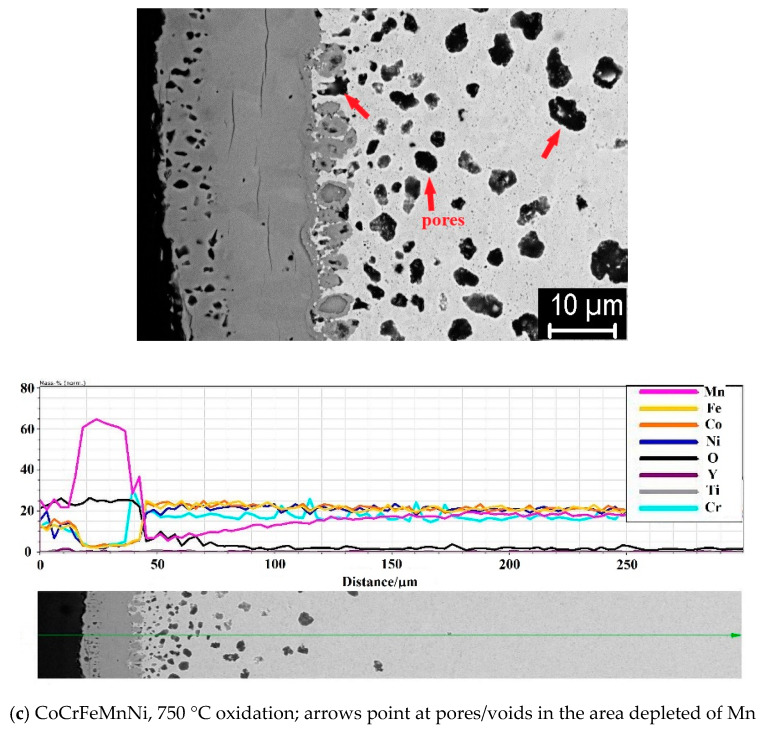
SEM images (backscatter mode) of the oxide scales coupled with plots of elemental distribution in the oxide scale and the adjacent alloy region measured by GDOES. The actual concentration of the element is multiplied by the factor described in the legend. The results refer to 150 h of oxidation at 750 °C.

**Figure 6:**
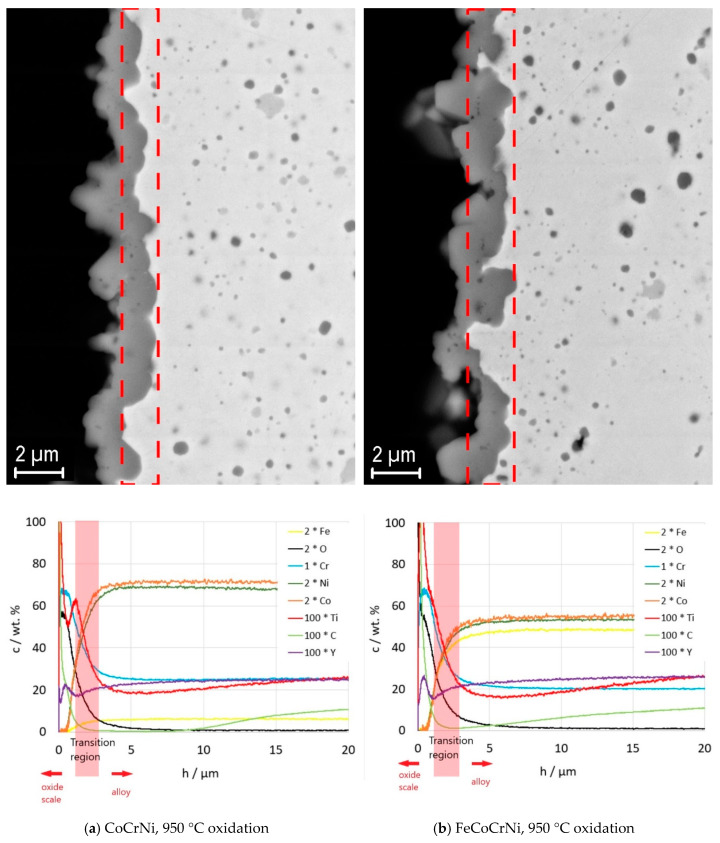
SEM images (backscatter mode) of the oxide scales coupled with plots of elemental distribution in the oxide scale and in the adjacent alloy region measured by GDOES. The actual concentration of the element is multiplied by the factor described in the legend. The results refer to 150 h of oxidation at 950 °C.

With increasing thickness, the oxide scale protrudes into the alloy and the alloy/scale interface becomes very rough (Figure 5 and Figure 6). Therefore, to interpret the GDOES results correctly, a transition region was marked in the plots corresponding to the regions marked in the respective micrographs above (Figure 5 and Figure 6). Due to the jagged interface, the results of the elemental analysis from the transition region include superposed information from the oxide and the alloy. Figure 5 shows the microstructure of the oxide scales and a plot of the elemental distribution in the oxide scales and the alloys underneath the scales after 150 h of oxidation at 750 °C. According to the results, the oxide scale consists mainly of chromium, oxygen, and carbon. There is no chromium-depleted layer formed underneath the scale, suggesting that the alloy provides a sufficient chromium supply for the scale formation up to the tested oxidation time. Both Y and Ti participate in the oxide scale formation. There is a sharp decrease in Y and Ti towards the surface in the oxide scale grown on CoCrNi at 750 °C (Figure 5a), whereas a Ti peak and an increased amount of Y is found in the scale grown on FeCoCrNi near the oxide surface (Figure 5b). The increase in Ti in the scale on FeCoCrNi correlates with the decrease in Cr. Ti was reported to have a good solubility in Cr_2_O_3_ [34]. The diffusion of Ti though the Cr_2_O_3_ scale was confirmed also on the scales grown on Ni-Cr alloys, resulting in the formation of a thin TiO2 outer layer [35,36,37]. Comparing the results for CoCrNi with FeCoCrNi (Figure 5), it is apparent that the addition of Fe into CoCrNi promotes Y and Ti diffusion through the Cr_2_O_3_ scale. Unlike Y, Ti was, however, confirmed to have properties detrimental to the oxidation resistance [36,37,38]. This fact must be considered and negotiated if needed with the design of the alloy’s temperature stability and irradiation damage resistance. 

A significant difference was observed in the microstructural evolution of the alloy adjacent to the oxide scale. The scale on the CoCrFeMnNi alloy consist mainly of Mn-based oxides (Table 2), and is relatively thick compared to the Cr_2_O_3_ scale on the CoCrNi and FeCoCrNi alloy. The fast and continuous diffusion of Mn out of the CoCrFeMnNi alloy led to the formation of subsurface porosity (Figure 5c), whereas no porosity was evolved for CoCrNi and FeCoCrNi. The area depleted of Mn correlates well with the porous area. More pores were developed in the area with a lower concentration of Mn.

**Figure 7:**
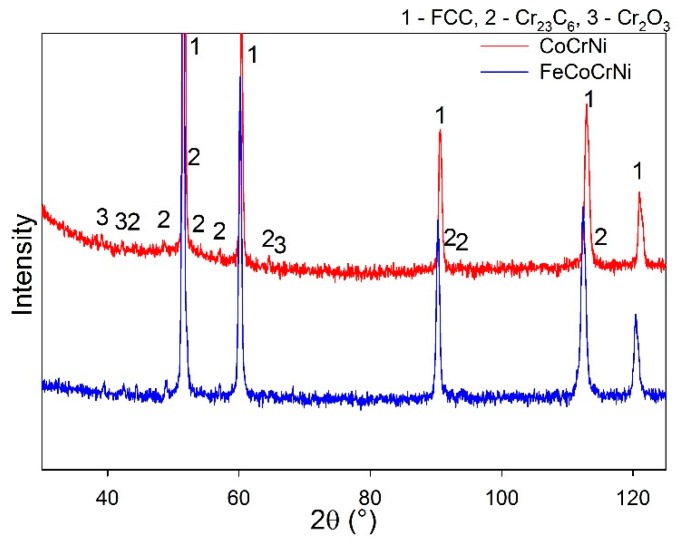
Phase analysis on the CoCrNi and FeCoCrNi alloys after 150 h oxidation at 950 °C. The analysis was performed in the area below the oxide scale outside the decarburized region.

The scale grown at 950 °C (Figure 6) consists of chromium, oxygen, carbon, and significantly larger amounts of titanium and yttrium than after oxidation at 750 °C. For both alloys, there are two Ti peaks (for FeCoCrNi it is less apparent, however, as demonstrated by the change in the Ti curve slope in the oxide scale), one with the maximum at the surface of the scale; the second is located within the transition region. The second Ti peak suggests that the oxide particles are not inert, even in the adjacent alloy, and experience changes induced by the oxidation process. Two explanations can be immediately considered: either the titanium diffuses out of the oxide particles or the particles dissolve and release all their constituents. However, Figure 6 suggests that the process might be even more complex. Since the drop in the concentration of Ti precedes the drop of Y (see the Y and Ti profiles in the alloys in Figure 6a,b), it might include Ti diffusion out of the oxide particle and the subsequent dissolution of yttrium-rich oxide. The addition of Fe into CoCrNi leads to the formation of a scale with a larger thickness variation, higher Ti and Y content, and a deeper Ti-depleted region in the alloy. Further, at 950 °C the alloys experience major carbon depletion. However, the depleted region is significantly shallower for FeCoCrNi; CoCrNi is decarburized down to zero carbon content up to 10 microns in depth, whereas a rather gradual decrease in carbon is observed in FeCoCrNi, reaching the minimum close to zero concentration at the oxide scale/alloy interface. Cr_23_C_6_ particles are likely to be the source of carbon, which can be correlated with the appropriate peak vanishing from the XRD spectra after oxidation. An additional XRD analysis of the oxidized alloys was performed in the area well below the oxide scale (Figure 7; only the 950 °C test results are presented). It was found that Cr_23_C_6_ carbides are present in the area. Thus, the disappearance of the carbides underneath the oxide scale is likely related to the oxidation process. Consistently, Berthod et al. and Durham et al. report that Cr-carbides dissolution is an alternative mechanism for balancing the chromium loss in Ni-Cr alloys [39,40]. The excess of free carbon resulting from the carbide dissolution escapes through the oxide scale, where it can react with oxygen and form a carbon oxide gas [39]. This is consistent with our observation of an increasing amount of carbon towards the oxide scale/air interface (Figure 6a,b) and the absence of a chromium-depleted region in the alloy.

## 4. Summary and Conclusions

CoCrNi, FeCoCrNi, and CoCrFeMnNi alloys were prepared by mechanical alloying and Spark Plasma Sintering. The alloys consisted of an FCC Ni-based matrix with a Y-Ti-oxide particle dispersion and additional Cr_23_C_6_ and Cr_2_O_3_ phases. The oxidation behavior of CoCrNi and FeCoCrNi alloys was studied and compared to the oxidation behavior of CoCrFeMnNi. The analyses were focused on the phase, composition, and structure changes of the scales and alloys resulting from the oxidation. 

The equiatomic addition of Mn to FeCoCrNi leads to the formation of Mn-based oxide scales that do not provide protection against oxidation. At 950 °C, the fast oxide growth causes major spallation at the beginning of the oxidation period. The continuous and fast diffusion of the Mn from the alloy leads to the development of relatively large pores in the regions adjacent to the oxide scale. 

CoCrNi and FeCoCrNi develop a Cr_2_O_3_ scale during oxidation at 750 °C and 950 °C that has a protective character for the tested period of 150 h. The addition of Fe to CoCrNi increases the weight gain at 950 °C and promotes Y and Ti diffusion through the Cr_2_O_3_ scale at both temperatures. For both alloys, the oxide particles are not inert, even in the adjacent alloy, and experience changes induced by the oxidation process resulting in the release of Ti and, in later stages, of Y from the oxide particles. Thus, Ti and Y-depleted regions are formed and pronounced during the oxidation at 950 °C. Cr_23_C_6_ particles underneath the oxide scale are dissolved during the oxidation process. The excess of free carbon resulting from the carbide dissolution escapes through the oxide scale and the alloys experience major carbon depletion at 950 °C. However, the depleted region is significantly shallower for FeCoCrNi. No chromium-depleted region was developed after the oxidation at both temperatures. 

The major constituent elements and oxide particles resulting from the minor additions of Y and Ti form a complex interplay that influences not only the response to the mechanical loading but also the high temperature oxidation. This effect must be considered during material tailoring for the intended application.

## Figures and Tables

**Table 1:** Elemental composition of the sintered alloys in wt. %. Carbon and oxygen are not included in the analysis due to the low sensitivity of the X-ray fluorescence (XRF) detector.

(wt. %)	Co	Cr	Ni	Fe	Mn	Y	Ti	Ca	Al	Cl	Si	S
CoCrNi	33.04	29.91	32.99	3.21	-	0.19	0.34	0.06	-	<0.05	<0.19	<0.03
FeCoCrNi	25.33	21.93	24.83	26.69	-	0.19	0.33	0.08	0.30	<0.04	<0.25	<0.02
CoCrFeMnNi	20.09	18.15	19.63	21.36	19.68	0.20	0.29	0.06	0.30	<0.04	<0.17	<0.02

**Table 2:** Phase content of the alloys measured by XRD after sintering and in the oxide scales developed after air exposure at 750 °C and 950 °C. The analysis of CoCrFeMnNi after 750 °C oxidation includes only the top oxide layer due to the large scale thickness; no analysis is provided after 950 °C oxidation due to the massive spallation of the oxide scale.

**Before Oxidation, wt. %**					
	**FCC Nickel-based Matrix**	**Cr_23_C_6_**	**Cr_2_O_3_**	**Spinel Oxide Structure**	
CoCrNi	88.83	5.33	5.83	-	-
FeCoCrNi	91.17	4.95	3.89	-	-
CoCrFeMnNi	86.41	10.50	1.05	2.04	-
**After Oxidation at 750 °C, wt. %**					
	**FCC Nickel-based Matrix**	**Cr_23_C_6_**	**Cr_2_O_3_**	**Mn_3_O_4_**	**Mn_2_O_3_**
CoCrNi	72.71	-	27.29	-	-
FeCoCrNi	89.22	2.93	7.85	-	-
CoCrFeMnNi	-	-	-	12.03	87.97
**After Oxidation at 950 °C, wt. %**					
	**FCC Nickel-based Matrix**	**Cr_23_C_6_**	**Cr_2_O_3_**	**Mn_3_O_4_**	**Mn_2_O_3_**
CoCrNi	43.13	-	56.87	-	-
FeCoCrNi	43.04	-	56.96	-	-
CoCrFeMnNi	-	-	-	-	-
